# Olfactory dysfunction as potential biomarker in neurodegenerative diseases: a narrative review

**DOI:** 10.3389/fnins.2024.1505029

**Published:** 2025-01-07

**Authors:** Nicolas De Cleene, Katarína Schwarzová, Samuel Labrecque, Clancy Cerejo, Atbin Djamshidian, Klaus Seppi, Beatrice Heim

**Affiliations:** Department of Neurology, Medical University of Innsbruck, Innsbruck, Austria

**Keywords:** neurodegeneration, biomarker, olfaction, hyposmia, early detection

## Abstract

Neurodegenerative diseases represent a group of disorders characterized by progressive degeneration of neurons in the central nervous system, leading to a range of cognitive, motor, and sensory impairments. In recent years, there has been growing interest in the association between neurodegenerative diseases and olfactory dysfunction (OD). Characterized by a decline in the ability to detect or identify odors, OD has been observed in various conditions, including Alzheimer’s disease (AD), Parkinson’s disease (PD), Huntington’s disease (HD), and Amyotrophic Lateral Sclerosis (ALS). This phenomenon often precedes the onset of other clinical symptoms, suggesting its potential utility as an early marker or prodromal symptom of neurodegenerative diseases. This review provides a vast literature overview on the current knowledge of OD in PD, AD, ALS, and HD in order to evaluate its potential as a biomarker, particularly in the early and prodromal stages of these diseases. We summarize the most common methods used to measure olfactory function and delve into neuropathological correlations and the alterations in neurotransmitter systems associated with OD in those neurodegenerative diseases, including differences in genetic variants if applicable, and cater to current pitfalls and shortcomings in the research.

## 1 Introduction

Neurodegenerative diseases represent a group of disorders characterized by progressive degeneration of neurons in the central nervous system, leading to a range of cognitive, motor, and sensory impairments (Choonara et al., 2009). This prevalence of these diseases is globally one of the fastest growing, mainly attributed to an aging population (Rapp et al., 2015; Reeve et al., 2014). Finding reliable and cost-effective biomarkers to improve clinical diagnosis, increase accuracy of differential diagnosis, predict and monitor disease progression, and evaluate responses to therapeutic interventions, is highly needed for both clinical and research purposes.

In recent years, there has been growing interest in the association between neurodegenerative diseases and olfactory dysfunction (OD). Manifesting as hyposmia or anosmia, it has been observed in various neurodegenerative conditions, including Parkinson’s Disease (PD), Alzheimer’s Disease (AD), Amyotrophic Lateral Sclerosis (ALS), and Huntington’s Disease (HD), among others. This phenomenon often precedes the onset of other clinical symptoms, suggesting its potential utility as an early marker or prodromal symptom of neurodegenerative diseases (Marin et al., 2018).

The choice of method for assessing olfactory function and dysfunction is crucial for ensuring accurate diagnosis, consistent outcome reporting, and reliable tracking of olfactory changes over time. A significant limitation in the existing literature is the variability in the assessment techniques employed. Broadly, three main types of olfactory testing are commonly used: patient reported assessment, psychophysical olfactory assessment, electrophysiological studies, or magnetic resonance imaging.

Subjective testing can be performed using visual analogue scales or questionnaires. In patients reporting dysfunction, olfactory assessment should be undertaken. Psychophysical tests offer a more reliable evaluation of olfactory function compared to subjective methods. These tests involve presenting an olfactory stimulus, with the outcome depending on the patient’s response. As such, psychophysical testing requires the cooperation of a subject who can comprehend instructions and effectively communicate their choices to the examiner.

Table 1, adapted from Hummel et al. (2017), lists several of the psychophysical tests used for olfactory assessment and the olfactory components they measure. Multiple olfactory testing batteries are available. The most frequently used test batteries are the University of Pennsylvania Smell Identification Test (UPSIT) and the Sniffin’ Sticks test battery. The UPSIT tests smell identification only, using four booklets containing 10 odors each (Doty et al., 1984). The participant is asked to choose the correct answer from four possibilities. By contrast, the Sniffin’ Sticks test battery contains three different subscales: threshold, discrimination, and identification (Hummel et al., 1997). In this test battery, the threshold subscale consists out of 16 triplets, each containing one odor and two blank sticks. The sticks containing odor have different concentrations, thus making it possible to evaluate at which concentration the individuals start to perceive the odor. The discrimination subscale contains 16 triplets, each containing duplets of the same odor and an ‘odd’ one out, which the participant has to identify from the three sticks presented. The identification subscale is similar to the UPSIT, in which a stick is presented to the participant, who has to guess the correct answer based on four given possibilities.

**Table 1:** Psychophysical tests for olfactory assessment adapted from Hummel et al. (2017).

Psychophysical test	Assessed olfactory components
Sniffin’ Sticks	Threshold, discrimination, identification
Barcelona Smell Test (BAST-24)	Odor detection, identification, memory
Connecticut Chemosensory Clinical Research Center Test	Threshold, identification
T & T Olfactometer	Threshold, identification
Smell Threshold Test	Threshold
Olfactory Perception Threshold Test	Threshold
Snap & Sniff Olfactory Test System	Threshold
University of Pennsylvania Smell Identification Test (UPSIT)	Identification
Smell Diskettes Test	Identification
Cross-Cultural Smell Identification Test	Identification
Pocket Smell Test	Identification
San Diego Odor Identification Test (SDOIT)	Identification
Scandinavian Odor Identification Test	Identification
Odorized Marker Test	Identification
Open Essence	Identification
Brief Smell Identification Test (B-SIT)	Identification

Table 2 summarizes the usual testing procedure for each typically tested olfactory domain (identification, discrimination, and threshold). Many other tests and variants exist, which cater to various limitations of the original tests [for example the length of the full UPSIT with the Brief Smell Identification Test (Doty, 2001), a visual version of the UPSIT for non-verbal patients (Rami et al., 2007), or an adaptation of the testing procedure in general for use during the COVID19 pandemic (Dietz et al., 2020)] and to various cultures due to typical smells differing across cultures [for example, the Odor Stick Identification Test for the Japanese (Saito et al., 2003)].

**Table 2:** Description of olfactory tests.

Olfactory domain	Usual test procedure
Identification	The participant smells one common odor and must identify it from a multiple choice list. This procedure is repeated over several odors. Cultural variants of tests help in keeping the tested common odors culturally and statistically relevant.
Discrimination	The participant smells several (3 or more) odors in a row, all but one of which are identical, and must identify which odor was different from the other. This procedure is repeated over several odors.
Threshold / detection	Several dilution of a particular odorant are used and are presented to the participant in decreasing order of dilution (i.e., each successive dilution smells stronger than the previous one). The participant must identify from three or more choices (the others usually containing water) which one contains the dilution. The test stops when the participant correctly identifies the dilution twice in a row.

Olfaction can also be assessed in a less subjective way using electrophysiological studies. This includes electroencephalography (EEG) and electro-olfactography (EOG), which records generator potentials via an electrode placed in contact with the olfactory neuroepithelium. Since both EEG and EOG are event-related, precise control over the delivery of a known concentration of odorant is required, typically achieved with an olfactometer. This need for precise odorant delivery limits their practical application in clinical settings (Knecht and Hummel, 2004; Rombaux et al., 2012).

Many studies have shown a multitude of changes in the central olfactory system following smell loss using magnetic resonance imaging (MRI) on both functional and structural levels (Lee et al., 2020). Among the available MRI-based techniques, olfactory bulb morphometry stands out as a highly reliable method, with olfactory bulb height identified as the most accurate parameter for detecting dysfunction (AUC = 0.85) after age adjustment (Lee et al., 2020). Furthermore, atrophy of the olfactory bulb, characterized by flattening and thinning with the loss of its typical oval or J-shape, serves as a hallmark of olfactory impairment (Lee et al., 2020). Volumetric analysis of the olfactory bulb shows a strong correlation with olfactory function, though its use in routine clinical practice is limited by the labor-intensive nature of manual contouring (Chung et al., 2018; Lie et al., 2021). Structural imaging, using coronal T2-and T1-weighted sequences with 2 mm slice thickness, allows for detailed assessment of the olfactory bulbs and tracts (Lie et al., 2021). Additional sequences, such as gradient echo CISS, may help detecting associated abnormalities, including cerebrospinal fluid leaks (Lie et al., 2021). Functional MRI (fMRI) provides insights into brain responses to odor stimuli, though it remains primarily a research tool rather than a standard clinical diagnostic modality (Han et al., 2019). Similarly, diffusion tensor imaging (DTI) can evaluate white matter integrity in central olfactory regions, further enhancing our understanding of olfactory pathways (Han et al., 2019).

Many individuals with OD are unaware of smelling problems until tested; for example, about half of the PD patients tend to overrate their sense of smell (Leonhardt et al., 2019). Intriguingly, less than 10% of AD patients complained of worsening in olfaction during the early stage of the disease, while over 80% demonstrated a significant decline of olfactory function (Zou et al., 2016).

In this review, we provide a comprehensive overview examining olfactory dysfunction as a potential biomarker in neurodegenerative diseases. Methodologically, we perused the Google Scholar and PubMed databases, combining keywords relating to olfaction and olfactory dysfunction to those of the diseases themselves, and also performed cross-referencing on the selected papers. By elucidating the intricate relationship between olfactory dysfunction and neurodegeneration, we underscore the significance of olfactory assessment in the broader context of biomarker research and clinical management of neurodegenerative disorders.

## 2 Parkinson’s disease

Parkinson’s disease (PD) is the second most common neurodegenerative disorder, with recent numbers showing a steep increase in disease prevalence (Steinmetz et al., 2024), primarily caused by an ageing population (Reeve et al., 2014; Dorsey et al., 2018).

Neuropathologically, PD is characterized by the progressive degeneration of dopaminergic neurons in the substantia nigra and the accumulation of misfolded alpha-synuclein into intracellular inclusions known as Lewy bodies. According to the prion-like hypothesis, alpha-synuclein aggregates originate in specific regions, such as the enteric nervous system and the olfactory bulb, and spread to connected brain areas via intra-axonal transport (Poewe et al., 2017). These early sites of pathology correspond to prodromal symptoms of PD, such as constipation and hyposmia, which often manifest years before motor symptoms appear (Poewe et al., 2017). Post-mortem studies confirm significant neuronal loss in the olfactory bulb and associated areas, including the anterior olfactory nucleus, amygdala, piriform cortex, and orbitofrontal cortex (Pearce et al., 1995; Harding et al., 2002; Silveira-Moriyama et al., 2009). As alpha-synuclein pathology spreads, it eventually affects the substantia nigra, leading to the cardinal motor symptoms of bradykinesia, rigidity, and resting tremor, which form the basis of clinical PD diagnosis (Durcan et al., 2019; Hawkes, 2008; Postuma et al., 2015).

However, motor symptoms typically emerge after 35–45% of dopaminergic neurons in the nigrostriatal pathway are lost, underscoring the importance of identifying individuals at risk during the preclinical or early stages of the disease (Heng et al., 2023). Various nonmotor symptoms (NMS) can precede the onset of the classic motor features in PD. Most of these early NMS include olfactory dysfunction (OD), rapid eye movement sleep behavior disorder (RBD), constipation, and mood disturbances such as depression (Pont-Sunyer et al., 2015; Ross et al., 2008).

OD is one of the most prevalent NMS is PD, affecting approximately 75% of diagnosed patients (Chen et al., 2015). It is considered an independent risk factor for the disease (Chen et al., 2015), with an odds ratio of 4.18 and increasing if combined with RBD (Iranzo et al., 2021; Janssen Daalen et al., 2021). Studies have shown deficits across all olfactory domains—identification, discrimination, and threshold—in PD patients (Trentin et al., 2022). Longitudinal studies, such as the Parkinson’s Associated Risk Syndrome (PARS) study, have emphasized the utility of serial olfactory testing, as some individuals initially categorized as hyposmic revert to normosmic values upon follow-up (Vaswani et al., 2022). Interestingly, idiopathic hyposmia has been associated with a 5% annual reduction in dopamine transporter (DAT) binding, a rate comparable to early-stage PD, further supporting its role as a preclinical biomarker (Jennings et al., 2017).

OD is particularly valuable in distinguishing PD from other parkinsonian syndromes and tremor disorders: PD patients demonstrate lower scores in olfactory testing compared to individuals with vascular parkinsonism, essential tremor, or atypical parkinsonian syndromes (Katzenschlager et al., 2004; Krismer et al., 2017; Shah et al., 2008; Wenning et al., 1995). Furthermore, olfactory deficits are more pronounced in idiopathic PD compared to drug-induced parkinsonism (Kwak et al., 2024). The clinical implications of OD extend beyond PD diagnosis. Several studies have explored the relationship between olfactory impairment and motor progression in PD, though results remain heterogeneous (He et al., 2020; Ercoli et al., 2022).

Notably, patients with the akinetic-rigid motor PD subtype tend to perform worse in odor threshold tests compared to those with tremor-dominant PD. Longitudinal data suggest that hyposmic patients are at increased risk for motor complications, such as freezing of gait, and often require higher doses of dopaminergic therapy over time (Lee et al., 2021). A recent study compared the two subtypes and found that the akinetic-rigid subtype performs worse in terms of odor threshold compared to the tremor-dominant subtype, but the two subtypes do not significantly differ in the other two domains (Solla et al., 2022).

Cognitive impairment is also a frequent symptom in PD compared to the general population, being associated with the natural history of the disease (Aarsland et al., 2001; Lawson et al., 2016). Two recent studies also reported cognition to impact scores on the discrimination and identification domains of the Sniffin’ Sticks test battery (Elhassanien et al., 2021; Wang et al., 2024). A longitudinal study recruited a large drug-naïve cohort of PD patients, which were followed for a period between three and 10 years (Yoo et al., 2020). Their data showed anosmic patients to have a higher rate of conversion to dementia compared to hyposmic and normosmic participants, independently of age, sex, and baseline motor and cognitive functions.

Longitudinal studies from the Parkinson’s Progression Markers Initiative (PPMI) have shown that patients with worse olfactory function at baseline exhibit greater cognitive decline, higher tau/Aβ1-42 ratios (Fullard et al., 2016), and more pronounced cerebral atrophy in regions such as the parahippocampal gyri and transverse temporal gyrus (Kawabata et al., 2024).

Olfactory impairment also varies with genetic subtypes of PD. For example, patients with the LRRK2 G2019S mutation exhibit less severe OD compared to idiopathic PD (55.6% vs. 85.4%) (Saunders-Pullman et al., 2022), whereas those with the SNCA/p.A53T or PARKIN mutation often experience profound olfactory deficits (Koros et al., 2018; Wang et al., 2017). Interestingly, ethnic differences in olfactory impairment have also been observed, emphasizing the need for population-specific studies (Cohen et al., 2023).

It has been shown that patients with idiopathic RBD and olfactory loss are at high short-term risk to develop PD and Dementia with Lewy bodies (DLB) (Mahlknecht et al., 2015; Miyamoto and Miyamoto, 2022). More recently, a prospective study found a comparable prevalence of RBD and hyposmia in individuals with PD (El Otmani et al., 2023), with a higher relative percentage of individuals experiencing OD before time of PD diagnosis compared to RBD.

In summary, OD serves as a crucial biomarker for PD, with diagnostic, prognostic, and therapeutic implications. Its association with early pathology, motor progression, and cognitive decline underscores its clinical significance. Future research should focus on combining OD with other biomarkers, such as imaging and fluid-based assays, to refine early diagnosis and to better predict disease progression. As longitudinal studies like PPMI continue to provide valuable insights, olfactory testing is likely to play an increasingly integral role in the comprehensive assessment of PD.

## 3 Alzheimer’s disease

Alzheimer’s disease (AD) is a chronic, progressive neurodegenerative disorder and the leading cause of dementia worldwide, accounting for 60–80% of all dementia cases (Nichols et al., 2022; Alzheimer’s Association, 2018). This chronic, progressive neurodegenerative disorder is characterized by cognitive decline and memory loss, primary driven by hallmark neuropathological features: extracellular amyloid-*β* plaques (Glenner and Wong, 1984; Hardy and Higgins, 1992; Hardy and Allsop, 1991; Masters et al., 1985; Selkoe, 1991; Selkoe and Hardy, 2016) and tau neurofibrillary tangles (Goedert et al., 1991; Kosik et al., 1986) in the brain, resulting in neuronal and synaptic loss (Serrano-Pozo et al., 2011). In addition to these primary drivers, a range of pathophysiological mechanisms have been described, including decreased acetylcholine activity (Mesulam, 1986; Racchi et al., 2001; Wong et al., 1999), autophagy (Nilsson and Saido, 2014; Nixon et al., 2005), inflammatory response (Fiala et al., 1998), glutamate toxicity (Greenamyre et al., 1988), neurovascular mechanism (Wang et al., 2023), and mitochondrial dysfunction (Valla et al., 2006). Peng et al. (2023) provide an excellent up-to-date summary of all mechanisms.

OD is increasingly recognized as a significant and early indicator of AD, with evidence dating back to the 1970s (Waldton, 1974). Neuropathological studies suggest that olfactory-related brain regions, including the entorhinal and transentorhinal cortices, are among the first areas to exhibit AD pathology, as highlighted in Braak’s staging model (Braak and Braak, 1997). A greater decline in olfaction is linked to a faster accumulation of amyloid-β (Aβ) and tau in areas related to olfaction (Tian et al., 2022), and a reduction in odor identification correlates with altered cerebrospinal fluid biomakers (Lafaille-Magnan et al., 2017) and Aβ deposition measured using [^18^F]-flutemetamol PET imaging (A-PET) (Wang et al., 2023). Meta-analyses on olfactory structure volumetry (Jobin et al., 2021; Jobin et al., 2021) consistently reported progressive atrophy of olfactory structures, including the olfactory bulb and of the primary olfactory cortex across the AD continuum which is already significantly measurable in the Mild Cognitive Impairment (MCI) stage. In dementia-free older adults, significant correlations have been identified between OD and reduced volumes in these regions, emphasizing its relevance even before clinical cognitive symptoms emerge (Dintica et al., 2019). Additionally, OD not only precedes the onset of cognitive symptoms by several years (Yaffe et al., 2017), but is also associated with a faster cognitive decline (Dintica et al., 2019) and a higher conversion rate to dementia (Yaffe et al., 2017). For a comprehensive review, see Albers et al. (2015). However, one study has shown that the ε4 allele of the ApoE gene can also impact olfactory function independently of dementia conversion (Olofsson et al., 2010).

The earliest preclinical stage of the disease is the Subjective Cognitive Decline (SCD) (Jessen et al., 2014). It is a self-reported measure of impaired cognitive abilities when standard tests might not yet discern objective impairment. Neuroimaging findings in subjects with SCD are consistent with the pathological alterations in MCI and dementia due to AD (Wang et al., 2020), including structural changes in olfactory regions (Chen et al., 2022; Papadatos and Phillips, 2023). This corresponds to other findings that individuals with SCD are more likely to develop MCI (about 15%) and dementia (about 27%) (Mitchell et al., 2014). SCD was also found to be linked with an increased probability of AD-related biomarker abnormalities (Glodzik-Sobanska et al., 2007). Multiple studies have indicated that SCD individuals demonstrate a significant odor identification impairment compared to controls (Jobin et al., 2021; Sohrabi et al., 2009; Wang et al., 2021). Meta-analytic findings (Bouhaben et al., 2024; Jung et al., 2019; Rahayel et al., 2012; Silva et al., 2018) proposed that among the olfactory domains, identification appears to be the most altered in the AD continuum, and impaired olfactory identification was suggested to be a better predictor of cognitive decline than deficits in verbal episodic memory (Devanand et al., 2008).

MCI includes degradation in at least one cognitive domain while daily functioning remains intact (Petersen et al., 2001; Petersen et al., 1997). Individuals with MCI can be subdivided into two categories: amnestic MCI (aMCI) and non-amnestic MCI (naMCI) (Portet, 2006). Those with aMCI exhibit memory loss as a primary symptom, whereas individuals with naMCI primarily display impairment across other cognitive domains. One study indicates that decreased odor identification is associated with an increased risk of developing aMCI, while its relationship with naMCI remains less clear (Roberts et al., 2016). However, other research suggests that OD may be significantly associated with both MCI subtypes (Vyhnalek et al., 2015; Dong et al., 2023). Importantly, proportional relationships between cognitive impairment and odor identification deficits have been observed in individuals with MCI, with olfactory identification emerging as a robust marker for distinguishing individuals with MCI from healthy controls and for predicting progression to dementia (Touliou et al., 2021). Research has consistently highlighted odor identification as the most affected olfactory domain in AD (Bouhaben et al., 2024; Jung et al., 2019; Rahayel et al., 2012; Roalf et al., 2017). In general, independently from other factors such as age and MMSE score, odor identification impairment was found to be a predictor for dementia (Conti et al., 2013).

Several validated tests, such as the University of Pennsylvania Smell Identification Test (UPSIT), Brief Smell Identification Test, and the San Diego Odor Identification Test, focus exclusively on this domain, likely explaining the wealth of data in this area. By contrast, odor discrimination seems to be the most impaired domain in the healthy elderly population (Dintica et al., 2019; Doty and Kamath, 2014). Combining olfactory assessments with neuropsychological evaluations significantly enhances diagnostic accuracy. One study demonstrated that pairing odor identification tests with neuropsychological tests resulted in 100% sensitivity for predicting AD, with specificity remaining at 80–90% (Lojkowska et al., 2011). This suggests that the combination of the results of both olfactory and neuropsychological tests could be a viable screening tool for the risk of developing AD (Conti et al., 2013; Lojkowska et al., 2011; Tahmasebi et al., 2019; Woodward et al., 2017). However, further research is needed to determine if odor discrimination or identification tests or both are better tools in predicting cognitive decline in people with early cognitive symptoms of AD including MCI (Audronyte et al., 2023; Sohrabi et al., 2012).

The integration of olfactory testing with other biomarkers has long been suggested to improve predictive accuracy. As early as 2008, a combination of odor identification testing, hippocampal and entorhinal cortex volumetry, cognitive assessments, and functional questionnaires was proposed as a multidimensional approach to assess the risk of conversion from MCI to AD (Devanand et al., 2008). Recent studies have supported the use of functional MRI (fMRI) alongside odor identification testing for early detection of AD. As a further multidimensional assessment for early AD, the joint utilization of UPSIT and fMRI has also been proposed (Zhu et al., 2023). Moreover, the combined impairments in odor identification and episodic memory have been linked to early changes in medial temporal lobe structures, which play a central role in both memory and olfactory processing. These findings suggest that olfactory dysfunction reflects broader neurodegenerative processes and highlight its relevance as a biomarker in the early stages of AD (Chen et al., 2022; Papadatos and Phillips, 2023; Conti et al., 2013).

The exact combination of tests that should be employed in the screening process remains insufficiently researched. Some findings indicate that a combination of episodic and semantic memory tests can distinguish individuals with aMCI and early AD from those experiencing normal aging (Spaan, 2016). Conti et al. (2013) demonstrated that the MCI olfactory-impaired group exhibited similar levels of global cognition (measured by the MMSE) to the MCI olfactory-normal group at baseline. However, a significant difference was observed in episodic verbal memory. Mi et al. (2023) have also shown that while the MMSE and MoCA scores do tend to correlate with olfaction scores, the degree of significance and scale of the correlation varies greatly from one cognitive group (healthy, MCI, AD) to the next. The combination of both odor identification and episodic memory impairments could be linked to early changes in medial temporal lobe structures. These structures are affected early in the AD continuum (Chen et al., 2022; Papadatos and Phillips, 2023; Conti et al., 2013) and are believed to be responsible for both memory and olfactory identification processes. Complementing research in this area would therefore be appropriate.

With almost 150 investigational agents for AD being tested in clinical trials as of Cummings et al. (2022), most of which being intended for the earlier stages of the disease, the need for early, inexpensive, accessible, and reliable biomarkers to predict the risk of dementia steadily increases. Understanding the role of olfactory dysfunction in the AD continuum not only aids in early diagnosis but also informs intervention strategies that could potentially slow or even prevent progression to symptomatic stages of the disease. Further research into the optimal combination of olfactory, cognitive, and imaging biomarkers is essential to advance screening and treatment efforts in this rapidly evolving field.

## 4 Amyotrophic Lateral Sclerosis

Amyotrophic Lateral Sclerosis (ALS) or Motor Neuron Disease, is a neurodegenerative disease of the upper motor neurons in the cortex and lower motor neurons in the brainstem and spine (Brooks, 1994; Shefner et al., 2020). This degeneration leads to muscle denervation, causing progressive weakness of voluntary muscles and progresses to respiratory difficulties and dysphagia (Fujimura-Kiyono et al., 2011; Lahrmann et al., 2003). The disease is less prevalent compared to AD and PD, though recent data showed an increase in incidence, explained by globally changing demographics such as aging (Longinetti and Fang, 2019). The disease presents with a highly heterogeneous phenotype, including variability in the age of onset. However, the prognosis remains poor, with most patients succumbing to respiratory failure within two to 5 years following diagnosis (Masrori and Van Damme, 2020).

Over 50% of ALS patients exhibit MCI or executive dysfunction, often mimicking features of Frontotemporal Dementia (FTD), with specific language difficulties, especially word naming, orthographic processing and syntactic and grammatical processing (Pinto-Grau et al., 2021; Strong et al., 2017). In fact, 10–15% of ALS patients show prominent comorbid FTD features (Phukan et al., 2007). The overlapping clinical and genetic spectrum of ALS and FTD is reflected in their shared pathology: TDP-43-positive neuronal inclusions are a hallmark feature of both diseases, reinforcing their classification as part of the TDP-43 proteinopathy continuum (Gao et al., 2018; Tan et al., 2017). Current clinical challenges in ALS are early disease identification, as diagnosis is often delayed due to mimicking disorders (Štětkářová and Ehler, 2021).

While OD in ALS has been observed for decades, the literature on this topic remains limited. One imaging study has identified an association between OD and atrophic changes in the bilateral medial orbital cortex and right hippocampus, regions integral to olfactory processing that receive input from the primary olfactory cortex (Masuda et al., 2021). Standard olfactory tests, such as the UPSIT, often rely on verbal responses, which can be challenging for ALS patients with cognitive or linguistic deficits. To address this, Rami et al. (2007) proposed adapting olfactory tests to include non-verbal methods, such as a parallel visual version, a picture-based version, and a picture-word matching test. These adaptations were later validated in a larger cohort by Pilotto et al. (2016), who demonstrated that ALS patients performed worse on both odor identification and discrimination tasks compared to healthy controls, except in cross-modal matching tests. Scores of ALS-FTD spectrum patients were generally worse compared to ALS patients without cognitive abnormalities (ALS-N), suggesting the use of olfactory testing in ALS to identify individuals with a more diffuse cortical involvement (Pilotto et al., 2016). They also found the 12-odor version of the Sniffin’ Sticks to yield a sensitivity of 71% and specificity of 100% in distinguishing ALS-N patients from those on the ALS-FTD spectrum. Notably, olfactory data was collected on average 20 months after symptom onset and 4.5 months after diagnosis, yet no association was observed between OD and disease duration (Lang et al., 2011). Therefore, the use of olfactory testing in the early clinical phase could yield an important advantage for early identification and prognosis, as individuals with ALS-FTD have a faster disease progression and lower survival time compared to ALS-N or with cognitive impairment and behavioral changes (Ye et al., 2021).

The underlying mechanisms of OD in ALS remain poorly understood. A postmortem study found TDP-43-positive inclusions to be most frequent in the hippocampus and least abundant in the olfactory bulb (Takeda et al., 2015). This concentration gradient suggests ALS pathology to start in the hippocampus, after which it spreads to the primary olfactory center, later reaching other regions and, at last, the olfactory bulb. This would explain why the worst olfactory scores are found in individuals with cognitive dysfunction or patients on the ALS-FTD spectrum, as the pathophysiology is more widely spread in the brain, assuming TDP-43-positive inclusions to be a pathophysiological explanation.

An intriguing observation is the relationship between olfactory performance and respiratory function. One study found that ALS patients with dyspnea exhibited lower olfactory scores, while those with preserved respiratory function (vital capacity >70% of the predicted value) performed comparably to healthy controls (Günther et al., 2018).

Data regarding olfaction in ALS is scarce, though important steps have been taken in the last decades. Further evaluation of an adapted version of olfactory tests should be conducted, addressing the specific cognitive changes in ALS patients. As disease prevalence is low due to the fast progression and death, multi-center studies are warranted to observe larger cohorts and collect more solid data.

## 5 Huntington’s disease

Huntington’s disease (HD) is an inherited neurodegenerative disease characterized by the gradual decline of cognitive, psychiatric, and motor functions (Illarioshkin et al., 2018). A mutation in the first exon of the huntingtin gene (HTT) on chromosome 4p16 causes an increase in the number of cytosine-adenine-guanine (CAG) repeats, which triggers the onset and progression of symptoms (The Huntington’s Disease Collaborative Research Group, 1993). HD exhibits various motor, behavioral, and cognitive signs and symptoms which, as in several other neurodegenerative disorders, often begin well before a clinical diagnosis is made (Bates et al., 2015).

OD has first been recognized as a nonmotor symptom of HD for about four decades (Moberg et al., 1987). A recent postmortem study (Highet et al., 2020) also showed the presence of aggregates of mutant Huntingtin protein (mHTT) in the olfactory bulbs of all 13 of their dissected HD patients, suggesting that the olfactory bulbs belong to the affected brain regions of HD. Despite this recent development, the idea of olfactory capability’s potential as an early biomarker in HD has been hypothesized at least as early as Bacon Moore et al. (1999). Nevertheless, there are still relatively few studies focusing on OD in HD.

The available evidence not only suggests that OD in HD becomes worse across all olfactory domains as the disease progresses (Bacon Moore et al., 1999; Amini et al., 2023; Nordin et al., 1995; Paulsen et al., 2008; Tabrizi et al., 2011), but also that mutation carriers without distinct motor symptoms exhibit impairments in odor identification as they approach the expected onset of motor symptom (Paulsen et al., 2008; Heim et al., 2020; Tabrizi et al., 2009). One study (Tabrizi et al., 2011) reported an acceleration of olfactory decline during later manifest stages, emphasizing the importance of studying OD in the early stages of the disease.

However, discrepancies exist regarding the specific olfactory domains affected in HD. Some studies report significant impairment on the identification and threshold domains only in manifest HD patients and not in premanifest mutation carriers compared to controls (Bylsma et al., 1997; Moberg and Doty, 1997). On the other hand, one study did not identify significant differences in threshold performance between the manifest group versus controls (Pirogovsky et al., 2007). Another study confirmed those findings in premanifest mutation carriers but did find significant impairment on the discrimination domain for that group (Larsson et al., 2006). More recent work, however, did show significant identification impairment in the group of premanifest mutation carriers compared to controls (Heim et al., 2020); it is argued that this difference in results from previous work comes from the higher sensitivity and specificity of newer tests.

Which olfactory domains are most affected in premanifest mutation carriers and manifest HD patients, and to what extent they are affected, therefore remains somewhat unclear, though the progressive dysfunction on the identification domain across the healthy-asymptomatic-manifest spectrum is generally shown in all studies. Manifest patients perform worse than controls in terms of discrimination (Amini et al., 2023; Nordin et al., 1995), but this is only partly true for the premanifest group (Larsson et al., 2006). In terms of threshold, manifest patients perform worse than both other groups (Bacon Moore et al., 1999; Amini et al., 2023; Nordin et al., 1995), but the premanifest and healthy groups do not significantly differ from one another (Pirogovsky et al., 2007; Larsson et al., 2006). In any case, more studies involving an asymptomatic cohort are needed, as data is generally scant.

The research on OD in HD, however, is, like the previously discussed diseases, subject to methodological limitations. While many of the studies at least partly used validated olfaction tests such as the Sniffin’ Sticks or the UPSIT (Amini et al., 2023; Nordin et al., 1995; Paulsen et al., 2008; Tabrizi et al., 2011; Heim et al., 2020; Tabrizi et al., 2009; Bylsma et al., 1997; Moberg and Doty, 1997), which have also often been compared with one another in many studies [e.g., (Campabadal et al., 2019; Hugh et al., 2015; Lawton et al., 2016; Wolfensberger et al., 2000)], others did not and instead used seldom used test batteries (Moberg et al., 1987; Bacon Moore et al., 1999; Pirogovsky et al., 2007; Larsson et al., 2006). Moreover, different olfactory domains were tested across the different studies. While the definition of staging of the disease varied across the different studies, the adoption of the newly developed HD-ISS (Tabrizi et al., 2022) should enable data standardization across future studies. All these inconsistencies make it difficult to compare results on OD in HD between studies. In addition, some of the few studies of OD in HD do not have OD as their focus (Paulsen et al., 2008; Tabrizi et al., 2011; Tabrizi et al., 2009). Rather, olfactory ability, limited to the identification domain, is one of a great many topics covered, which, considering the quantity of test data reported in those papers, is understandably given very little place and analysis.

In summary, while current studies on living HD patients do not provide conclusive evidence that OD is a reliable biomarker for HD, they do highlight its significance as a relevant aspect of the condition. OD is particularly intriguing in premanifest patients, as it may serve as an early indicator of neurodegeneration. This underscores the need for further research with a focus on all olfactory domains, using standardized methodologies to explore its potential as a biomarker more thoroughly.

## 6 Conclusion

Extensive data regarding olfactory dysfunction in neurodegenerative disorders has been published over the last decades, both in individuals at risk as well as in patients with the disease. Table 3 presents and summarizes the findings discussed above for all three olfactory domains and each disease and provides important points of the clinical relevance of OD in those diseases.

**Table 3:** Summary of olfactory dysfunction per olfactory domain and disease.

Olfactory domain	Parkinson’s disease	Alzheimer’s disease	Amyotrophic lateral sclerosis	Huntington’s disease
Identification	Decreased performance compared to controls and compared to DIP. Performance impacted by cognition.	SCD: Decreased performance compared to controls. MCI (both aMCI and naMCI): Decreased performance compared to controls. Most affected domain in AD continuum.	Decreased performance of both ALS-FTD and ALS-N compared to controls, with ALS-FTD performing worse than ALS-N.	Manifest: decreased performance compared to asymptomatic carriers as well as controls. premanifest: decreased performance compared to controls.
Discrimination*	Decreased performance compared to controls and compared to DIP. Performance impacted by cognition.	Most impaired in the healthy elderly population.	Decreased performance of both ALS-FTD and ALS-N compared to controls, with ALS-FTD performing worse than ALS-N.	Manifest: decreased performance compared to controls. premanifest: partly decreased performance compared to controls.
Threshold*	Decreased performance compared to controls	*Too little information, more research is needed.*	*Too little information, more research is needed.*	Manifest: decreased performance compared to asymptomatic carriers as well as controls. premanifest: no significant difference from controls.
Clinical relevance of olfactory dysfunction on disease progression	Increased risk of developing freezing of gait. High short-term risk of PD if coupled with iRBD. Differentiator of PD and related syndromes. Anosmia leads to higher conversion to dementia. Some genetic variants are less inclined to OD than iPD, and some are more.	Greater OD is associated with faster accumulation of Aβ. OD and structural changes in olfactory regions precede cognitive symptoms by several years. Anosmia leads to a higher conversion to dementia and to a more rapid cognitive decline. Decreased odor identification leads to an increased risk of aMCI (but no correlation with naMCI). Identification impairment is a predictor for dementia. Combining olfactory and neuropsychological tests could be a viable screening tool for AD risk.	Olfactory testing can be used to distinguish ALS-FTD and ALS-N. No association between olfactory dysfunction and disease duration.	The olfactory bulbs belong to the affected brain regions of HD. Identification impairment increases as asymptomatic mutation carriers near motor symptom onset.

As most studies focus solely on odor identification, the summary of these categories as discussed in the review may not have as high as statistical degree of confidence as for identification. Aβ, amyloid-beta; AD Alzheimer’s disease, ALS-FTD, Amyotrophic lateral sclerosis with frontotemporal dementia; ALS-N, Amyotrophic lateral sclerosis with normal cognition; DIP, Drug-induced Parkinsonism; HD, Huntington’s Disease; iRBD, Idiopathic REM sleep behavior disorder, (n (a)) MCI, (Non-(Amnestic)) Mild cognitive impairment; OD, Olfactory dysfunction; (i) PD, (Idiopathic) Parkinson’s disease; SCD, Subjective cognitive decline.

OD is prevalent in all neurodegenerative disorders, especially in PD and AD. The current data points favorable towards the application of olfactory dysfunction as a biomarker in neurodegenerative diseases, even showing sensitivity during the preclinical and prodromal phases in some disorders.

The current shift towards a biological definition in some of the diseases is important, urging future research to use the newly defined stages. Multicentric studies with large cohorts are currently performed, with more results being expected to be published in the next decade, offering much needed longitudinal data.
